# Tyrosine Kinase Inhibitors Do Not Promote a Decrease in SARS-CoV-2 Anti-Spike IgG after BNT162b2 Vaccination in Chronic Myeloid Leukemia: A Prospective Observational Study

**DOI:** 10.3390/vaccines10091404

**Published:** 2022-08-27

**Authors:** Seiichiro Katagiri, Daigo Akahane, Shunsuke Otsuki, Arisa Suto, Akiko Yamada, Tamiko Suguro, Michiyo Asano, Seiichiro Yoshizawa, Yuko Tanaka, Nahoko Furuya, Hiroaki Fujimoto, Seiichi Okabe, Moritaka Gotoh, Yoshikazu Ito, Akihiko Gotoh

**Affiliations:** Department of Hematology, Tokyo Medical University, 6-7-1 Nishishinjuku, Shinjuku-ku, Tokyo 160-0023, Japan

**Keywords:** chronic myeloid leukemia, tyrosine kinase inhibitors, BNT162b2 vaccine, anti-spike IgG

## Abstract

We performed a prospective observational study of chronic myeloid leukemia (CML) patients after anti-SARS-CoV-2 BNT162b2 vaccination (VC). In total, 32 CML patients with tyrosine kinase inhibitor (TKI) therapy, 10 CML patients with treatment-free remission, and 16 healthy subjects participated in the study. From April 2021 to September 2021, all cases (median age = 58 years) were vaccinated twice. Immunoglobulin G for SARS-CoV-2 spike protein (S-IgG) was measured at three timepoints (before the first VC, 1–5 weeks after the second VC (T1), and approximately 6 months after the second VC (T2)). S-IgG was not observed before the first VC in any participant. At T1, all cases had acquired S-IgG. There were no significant differences in S-IgG levels among groups. A paired sample comparison of median S-IgG titers between T1 and T2 in all groups showed a significant reduction in T2 S-IgG titers. There were no significant differences in S-IgG levels among groups. When all patients were analyzed, those aged ≥58 years had significantly lower S-IgG levels than those aged <58 years at T1. The BNT162b2 vaccine was highly effective in CML patients with or without TKIs, and S-IgG levels were as persistent as those in healthy individuals.

## 1. Introduction

Coronavirus disease 2019 (COVID-19), caused by severe acute respiratory syndrome coronavirus-2 (SARS-CoV-2), has spread across the world since 2019 and is still a major social and health problem [1]. Patients with hematologic malignancies often develop immunodeficiencies associated with the disease or use of therapeutic drugs, including chemotherapy and immunosuppressive agents, and are at very high risk of developing severe COVID-19 [2,3]. SARS-CoV-2 vaccines, including mRNA vaccines, are effective at preventing the onset and disease severity of COVID-19 [4,5]. Serological diagnosis is very important for diagnosing COVID-19 and evaluating the efficacy of SARS-CoV-2 vaccination [6,7]. Because most SARS-CoV-2 vaccines are designed to induce an immune response against the SARS-CoV-2 spike protein, many clinical research studies have used immunoglobulin G for SARS-CoV-2 spike protein (S-IgG) to determine vaccine efficacy [8,9,10]. It was recently reported that SARS-CoV-2 vaccine efficacy or efficacy against infectious and symptomatic diseases may be reduced 6 months after vaccination [9,11].

Tyrosine kinase inhibitors (TKIs) have dramatically improved the prognosis of patients with chronic myeloid leukemia (CML) [12]. Recently, it has become possible to discontinue TKIs in some CML cases that have maintained deep molecular responses for longer than 1 or 2 years [13,14,15,16,17,18,19]. In addition, it was reported that immune functions related to NK cells, CD8-positive T cells, and regulatory T cells affected the suppression of disease recurrence after the discontinuation of TKIs [20,21,22].

Recent reports showed that the BNT162b2 vaccine (Pfizer-BioNTech) was effective for antibody acquisition, even in CML cases administered TKIs [23,24]. However, there are few data on the long-term maintenance of antibody titers. We evaluated the effects of two vaccinations with BNT162b2 in CML patients, including those that had discontinued TKIs, up to 6 months after vaccination.

## 2. Materials and Methods

### 2.1. Patients

The subjects were CML chronic phase (CML-CP) patients aged 18 years or older who visited Tokyo Medical University Hospital. Patients who continued TKI treatment (CML-TKIs) and those who discontinued TKIs and maintained treatment-free remission (CML-TFRs) were included. Healthy controls (HCs) were also enrolled in the study. Written consent to participate in the study was obtained when the participants were scheduled to receive the BNT162b2 vaccine. Those with a history of COVID-19 prior to participation in the study were excluded. From April 2021 to September 2021, all participants received two doses of the BNT162b2 vaccine at a 3-week interval.

### 2.2. Assessment of Serological Response

S-IgG was measured with ARCHITECT SARS-CoV-2 IgG II Quant (Abbott, IL, USA). Samples were considered negative for antibody titers <50 AU/mL. Measurements were planned at three timepoints: (1) before the first vaccination, (2) 1–5 weeks after the second vaccination (T1), and (3) approximately 6 months after the second vaccination (T2). Patients were assessed when they visited the hospital for regular visits for CML medical care. Participants who were positive for S-IgG before the first vaccination or from whom samples could not be collected at T1 were excluded from the analysis.

### 2.3. Presence or Absence of COVID-19

We confirmed whether COVID-19 was diagnosed at the medical institution 6 months after the second vaccination.

### 2.4. Statistical Analysis

The primary and secondary endpoints were serological responses at T1 and T2, respectively. The Mann–Whitney *U*-test was used to compare antibody titers between the two groups. The Kruskal–Wallis test was used to compare the median age, median duration from second vaccination to T1/T2, and median antibody titers among the three groups (CML-TKI, CML-TFR, and HC). Median antibody titers by TKI were also compared using the Kruskal–Wallis test. The Wilcoxon test was used to perform paired sample comparisons of median antibody titers between T1 and T2. Spearman’s rank correlation coefficient was used to assess the relationship between two variables. A *p*-value < 0.05 was considered statistically significant. All statistical analyses were performed with Prism version 9.3.1 (GraphPad Software, San Diego, CA, USA).

## 3. Results

### 3.1. Characteristics of Participants

Thirty-five CML-TKIs, eleven CML-TFRs, and sixteen HCs participated in the study. No patients were positive for S-IgG at T0. At T1, appropriate samples were collected from 32 CML-TKIs, 10 CML-TFRs, and 16 HCs, respectively (median age = 58 years). Four cases were not scheduled for an outpatient visit at T1. Appropriate samples were collected from 27 CML-TKIs, 7 CML-TFRs, and 16 HCs at T2. Reasons why samples could not be collected from eight cases at T2 were as follows: one case developed COVID-19, one case discontinued TKI, one case died because of another disease, two cases were vaccinated for the third time, and three cases lacked an outpatient visit at T2. Patient backgrounds are shown in Table 1. There were no significant differences in age among the three groups. The duration from the second vaccination to T1 or T2 was significantly shorter in the HC group than in the CML-TKI and CML-TFR groups, respectively. No cases were diagnosed with COVID-19 in the HC and TKI-TFR groups. Only one case of CML with TKI therapy was diagnosed with COVID-19.

### 3.2. Serological Responses 1–5 Weeks after the Second Vaccination (T1)

**Figure 1:**
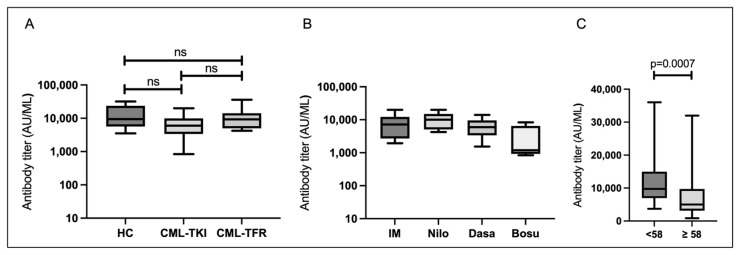
Serological responses 1–5 weeks after the second vaccination. Boxes show the interquartile range; the center line indicates the median; the whiskers indicate maximum and minimum values. (**A**) SARS-CoV-2 spike protein immunoglobulin G (S-IgG) titers in healthy controls (HCs) (n = 16), chronic myeloid leukemia patients who continued TKI treatment (CML-TKIs) (n = 32), and those who discontinued TKIs and maintained TFR (CML-TFRs) (n = 10). (**B**) S-IgG titers among those administered different TKIs in the CML-TKI group. (**C**) S-IgG titers by age (<58 and ≥58 years; n = 58). Abbreviations: ns, not significant; IM, imatinib; Nilo, nilotinib; Dasa, dasatinib; Bosu, bosutinib.

At T1, all cases had acquired S-IgG (median S-IgG level: 7596 AU/mL). The median S-IgG titers in the HC, CML-TKI, and CML-TFR groups were 9437, 5987, and 9238 AU/mL, respectively. The CML-TKI group tended to have lower antibody titers than the other groups, but there were no statistically significant differences among the three groups (Figure 1A; *p* = 0.1126). In the CML-TKI group, the median S-IgG titers were 8158 AU/mL for imatinib, 10,858 AU/mL for nilotinib, 6578 AU/mL for dasatinib, and 3216 AU/mL for bosutinib, with no statistical differences between the TKIs (Figure 1B; *p* = 0.06). When all participants were analyzed, S-IgG titers at T1 correlated with age (Supplemental Figure S1A; *p* = 0.0014). Those aged 58 years or older had significantly lower S-IgG levels than those aged <58 years (Figure 1C; *p* = 0.0007). The S-IgG titer at T1 did not correlate with duration from the second vaccination to T1 (Supplemental Figure S1B; *p* = 0.2945). There were no significant differences in the S-IgG titers at T1 by sex in none of the participants (*p* = 0.641).

### 3.3. Serological Responses Approximately 6 Months after the Second Vaccination (T2)

**Figure 2:**
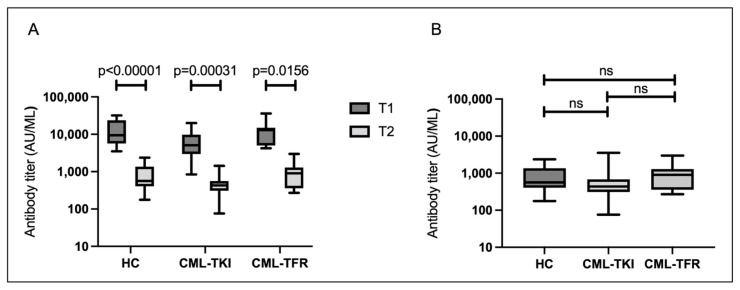
Serological responses approximately 6 months after the second vaccination. Boxes show the interquartile range; the center line indicates the median; and the whiskers indicate maximum and minimum values. (**A**) Paired sample comparison of median SARS-CoV-2 spike protein immunoglobulin G (S-IgG) titers between T1 and T2 in the three groups. (**B**) S-IgG titers in healthy controls (HCs) (n = 16), in chronic myeloid leukemia patients who continued TKI treatment (CML-TKIs) (n = 27), and those who discontinued TKIs and maintained TFR (CML-TFRs) (n = 7). Abbreviations: ns, not significant.

At T2, there were no cases in which S-IgG was negatively converted (median S-IgG level: 517 AU/mL). The median S-IgG titers between T1 and T2, according to the Wilcoxon test, were significantly reduced at T2 in all three groups (Figure 2A). The median S-IgG titers at T2 in the HC, CML-TKI, and CML-TFR groups were 566, 435, and 904 AU/mL, respectively. There were no significant differences in S-IgG levels among the three groups (Figure 2B; *p* = 0.1293). When all participants were analyzed, the S-IgG titers at T2 correlated with age (Supplemental Figure S1C; *p* = 0.0132). The S-IgG titers at T2 did not correlate with duration from the second vaccination to T2 (Supplemental Figure S1D; *p* = 0.9553). There were no significant differences in the S-IgG titers at T2 by sex in none of the participants (*p* = 0.247).

### 3.4. Molecular Response of CML at T2 in CML-TKI and CML-TFR Groups

At T2, five patients in the CML-TKI group changed their molecular response from pre-vaccination. Four patients achieved a deeper molecular response, and one showed a change from undetectable to MR4.0 (Supplemental Table S1). All patients in the CML-TFR group maintained their molecular response from pre-vaccination to T2.

## 4. Discussion

SARS-CoV-2 vaccines may be less effective in patients with hematopoietic malignancies, especially those using rituximab or Bruton’s tyrosine kinase inhibitors [25,26,27], those with multiple myeloma who receive four lines or more of treatment regimens, or patients with lower-than-normal gamma globulin levels [28]. Furthermore, lower anti-SARS-CoV-2 antibody titers 3 months after a second mRNA-based vaccination were observed in acute myeloid leukemia cases receiving maintenance therapy and myelodysplastic syndromes compared with healthy subjects [29].

Currently, four TKIs (imatinib, nilotinib, dasatinib, and bosutinib) are used as 1st line therapy for new CML-CP cases [30]. These TKIs inhibit BCR-ABL1 and have an off-target effect that inhibits other kinases, such as c-KIT, PDGFR, and SRC. It was reported that this off-target effect affects the immune system. Lavallade et al. reported that TKIs, through the off-target inhibition of kinases such as Btk or PLC-g2, which are important for B-cell signaling, may reduce memory-B-cell frequencies in CML [31]. It was also reported that the use of TKIs reduced plasma immunoglobulin levels [32,33].

TKIs may also affect the efficacy of vaccines other than SARS-CoV-2 vaccines. It was reported that humoral immunity to a pneumococcal vaccine might be impaired in CML patients receiving imatinib or dasatinib and that antibody acquisition after live, attenuated vaccines might be poor in pediatric CML patients receiving imatinib [31,34]. However, the efficacy of the influenza vaccine in CML patients receiving imatinib or dasatinib was higher than in patients with B-cell malignancies or those receiving allogeneic stem-cell transplants, and there were no significant differences from healthy subjects [35]. One of the mechanisms by which TKIs inhibit the vaccination effect is thought to be the suppression of memory B cells via the off-target effect of TKIs as mentioned above [31]. TKIs were also shown to inhibit T-cell proliferation in vitro [36,37]. However, T-cell responses to H1N1 vaccine or pneumococcal vaccine were maintained, even in CML patients with TKI therapy [31,35].

Our study reports the long-term follow-up data for CML patients vaccinated with BNT162b2. After two vaccinations, there were no significant differences in S-IgG titers between the HC, CML-TKI, and CML-TFR groups. In addition, no differences in S-IgG titers were observed for each TKI. This suggests that TKIs have a limited effect on the antibody acquisition induced by the BNT162b2 vaccine. Furthermore, after 6 months, the S-IgG antibody titers were lower than those after the second vaccination in all subjects, although there were no significant differences among the three groups. This suggests that the continuous administration of TKIs did not cause a decrease in antibody titers. Of all the study cases, only one subject in the CML-TKI group was diagnosed with COVID-19. After the observation period of this study, two participants in the CML-TKI group and two in the HC group were diagnosed with COVID-19 infection by June 2022. In this study, patient age influenced antibody titers after the second vaccination, with significantly lower titers in patients older than the median age of 58 years (calculated for all study participants).

In addition, vaccination might affect tumor immunity and influence the molecular responses of CML patients. Indeed, in our cohort, only 5 out of 42 patients showed a change in response before and after vaccination, with 4 patients having a deeper molecular response and only 1 patient showing a worsened response. It is especially noteworthy that all patients in the CML-TFR group showed no changes in molecular responses. However, because this analysis contained a low number of cases, this point should continue to be examined with more cases.

There were some limitations in the present study. Neutralizing antibodies were not measured in our study; however, the ARCHITECT SARS-CoV-2 IgG II Quant used here was confirmed to have a high correlation with the neutralizing antibody assay in various studies [38,39]. In addition, because only antibodies against the SARS-CoV-2 spike protein were examined, we could not exclude the possibility of asymptomatic infected individuals. Furthermore, this analysis did not evaluate T-cell immunity, which should be investigated in the future. Harrington et al. reported a SARS-CoV-2-specific CD4+ T-cell response in an analysis of CML patients who received a single dose of the BNT162b2 vaccine [24]. How et al. reported that patients with myeloproliferative neoplasms, including CML, were significantly less likely to exhibit T-cell responses than HCs, and they considered that this might have been associated with the older age of the myeloproliferative neoplasms group compared with the control group [40].

Recently, a third SARS-CoV-2 vaccination has been provided. However, the efficacy of a third vaccination in CML patients is unknown. A third BNT162b2 vaccination was reported in patients with hematologic malignancies who failed or lost seroconversion after the second vaccination [41]. In that report, 33.8% of patients responded to the third vaccination. In our present study, CML patients had the same vaccine effect as the HCs; thus, it is expected that a third SARS-CoV-2 vaccination of CML patients with BNT162b2 will be effective.

## 5. Conclusions

Our study showed that the BNT162b2 vaccine was highly effective in CML patients with and without TKIs, and that S-IgG levels were as persistent as those in healthy individuals. These results are expected to aid the treatment of CML in the era of the COVID-19 pandemic.

## Figures and Tables

**Table 1:** Participants’ characteristics.

	HC (*n* = 16)	CML-TKI (*n* = 32)	CML-TFR (*n* = 10)
Age (years), median (range)	45 (30–92)	62 (29–81)	61.5 (47–80)
Sex (male/female)	8/8	20/12	6/4
TKI (administration or discontinuation)			
Imatinib		6	7
Nilotinib		9	2
Dasatinib		12	1
Bosutinib		5	0
TKI dose (mg), median (range)			
Imatinib		350 (100–600)	
Nilotinib		600 (150–600)	
Dasatinib		60 (20–100)	
Bosutinib		200 (100–400)	
Duration of TFR (months), median (range)			68.5 (31–130)
CML response according to ELN criteria			
Non-MMR		1	0
MMR		3	0
MR4.0		11	1
MR4.5		2	2
Undetectable		15	7
Duration from 2nd VC to T1 (days)	27 (15–30) *	17.5 (7–35)	25.5 (8–31)
Duration from 2nd VC to T2 (days)	204 (190–215) *	174 (151–207)	174 (164–183)

Abbreviations: CML, chronic myeloid leukemia; TKI, tyrosine kinase inhibitor; TFR, treatment-free remission; ELN, European leukemia net; MMR, major molecular response; VC, vaccination; T1, within 1–5 weeks after second VC; T2, around 6 months after second VC. * *p* < 0.05.

## Data Availability

Not applicable.

## Supplementary Materials

The following supporting information can be downloaded at: https://www.mdpi.com/article/10.3390/vaccines10091404/s1, Figure S1: Correlation between SARS-CoV-2 spike protein immunoglobulin G titers and age, or duration from the second vaccination to T1/T2, Supplemental Table S1: Details of changes in molecular responses from pre-vaccination to T2.
