# Supplementary material for: Tyrosine Kinase Inhibitors Do Not Promote a Decrease in SARS-CoV-2 Anti-Spike IgG after BNT162b2 Vaccination in Chronic Myeloid Leukemia: A Prospective Observational Study
Source: Vaccines (Basel). 2022 Aug 27;10(9):1404. doi: 10.3390/vaccines10091404 (PMC9501552; doi:10.3390/vaccines10091404)
Supplement: Supplementary file 1 [file vaccines-10-01404-s001.zip › vaccines-1860018-supplementary.pdf]

Supplemental materials.

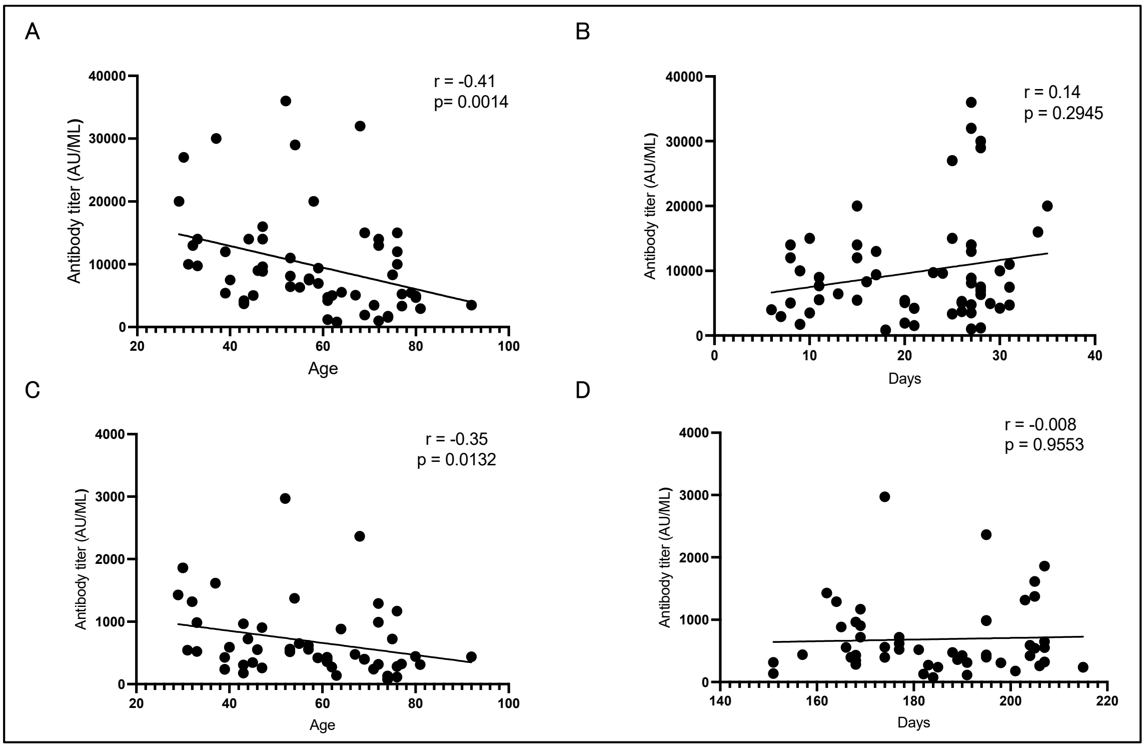

Figure S1: Correlation between SARS-CoV-2 spike protein immunoglobulin G titers and age, or duration from the second vaccination to T1/T2. (A) Correlation between SARS-CoV-2 spike protein immunoglobulin G (S-IgG) titers and age in all participants 1–5 weeks after the second vaccination (T1) (n=58). (B) Correlation between S-IgG titers and duration from the second vaccination to T1 in all participants (n = 58). (C) Correlation between S-IgG titers and age in all participants approximately 6 months after the second vaccination (T2) (n = 50). (D) Correlation between S-IgG titers and duration from the second vaccination to T2 in all participants (n = 50).

Supplemental Table S1

Details of molecular response change from pre-vaccination to T2

			Molecular response	
Participant's number	TKI	TKI dose (mg)	pre-vaccination	T2
#3	Dasatinib	70	non-MMR	MMR
#14	Dasatinib	70	MMR	MR4.0
#26	Nilotinib	600	MR4.0	MR4.5
#32	Nilotinib	600	MMR	MR4.0
#39	Imatinib	100	Undetectable	MR4.0

Abbreviations: TKI: tyrosine kinase inhibitor, MMR: major molecular response, T2: around 6 months after 2nd VC,
